# Rabies Vaccine Hesitancy and Deaths Among Pregnant and Breastfeeding Women — Vietnam, 2015–2016

**DOI:** 10.15585/mmwr.mm6708a4

**Published:** 2018-03-02

**Authors:** Huong T.T. Nguyen, Cuc H. Tran, Anh D. Dang, Huong G.T. Tran, Thiem D. Vu, Thach N. Pham, Hoang V. Nguyen, Anh N.K. Nguyen, Emily G. Pieracci, Duong N. Tran

**Affiliations:** ^1^National Institute of Hygiene and Epidemiology, Ministry of Health of Vietnam; ^2^Epidemic Intelligence Service, CDC; ^3^Poxvirus and Rabies Branch, National Center for Emerging and Zoonotic Infectious Diseases, CDC; ^4^International Cooperation Department, Ministry of Health of Vietnam; ^5^Hanoi Preventive Medicine Center, Ministry of Health of Vietnam.

Human rabies deaths are preventable through prompt administration of postexposure prophylaxis (PEP) with rabies immune globulin and rabies vaccine after exposure to a rabid animal (*1*); there are no known contraindications to receiving PEP (*1*,*2*). Despite widespread availability of PEP in Vietnam, in 2015 the Ministry of Health (MoH) received reports of pregnant and breastfeeding women with clinically diagnosed rabies. MoH investigated factors associated with these rabies cases. MoH found that, during 2015–2016, among 169 cases reported in Vietnam, two probable cases of rabies were reported in breastfeeding mothers and four in pregnant women, all of whom had been bitten by dogs. All six patients died. Three of the four pregnant women had cesarean deliveries. One of the three newborns died from complications believed to be unrelated to rabies; the fourth pregnant woman contracted rabies too early in pregnancy for the fetus to be viable. Two of the patients sought care from a medical provider or traditional healer; however, none sought PEP after being bitten. In each case, families reported the patient’s fear of risk to the fetus or breastfed child as the primary barrier to receiving PEP. These findings highlight the need for public health messaging about the safety and effectiveness of PEP in preventing rabies among all persons with exposures, including pregnant and breastfeeding women.

## Investigation and Results

Human rabies cases are reportable to MoH in Vietnam within 48 hours after clinical diagnosis, based on the World Health Organization’s probable case definition* (*1*). Probable cases of human rabies are investigated by MoH staff members from the National Rabies Control Program, using a standardized form to collect information on demographics, exposure, clinical symptoms, treatment, barriers to receipt of PEP, and outcomes. Although confirmatory laboratory testing is available, for various reasons including cultural barriers, hesitation by medical providers to collect specimens, and the cost of shipping specimens to the national laboratory, very few specimens are collected (*3*). Vietnam is committed to increasing accessibility of PEP throughout the country. Bite victims can receive care at any district or provincial medical center but are responsible for PEP-associated costs (approximately $153 USD for a full course, about equal to the average monthly salary in Vietnam) (*3*).

To assess risk factors for not receiving rabies PEP, investigation forms for probable rabies cases identified during 2015–2016 were reviewed. To evaluate the accessibility of PEP to the six pregnant or breastfeeding women with rabies, the distance and estimated travel time by automobile or motorized scooter (the primary mode of transportation) to district and provincial-level medical centers from the homes of the six patients were measured. The median age of the six pregnant or breastfeeding women with rabies was 27 years (range = 19–33 years), and all patients had attended at least junior high school (Table). The mean incubation period (from dog exposure to symptom onset) was 85 days (range = 49–126 days). The median interval from symptom onset to death was 2 days (range = 1–4 days).

**TABLE:** Selected characteristics, animal exposure, signs and symptoms, and treatment for six fatal rabies cases in pregnant and breastfeeding women — Vietnam 2015–2016

Characteristic	No.
**Education**	
Junior high school	5
Senior high school	1
**Dog bite**	6
**Status of dog at time of patient exposure**	
Normal*	2
Stray	3
Ill	1
**Dog rabies vaccination status**	
Yes	0
No	2
Unknown	4
**Bite location**	
Foot or leg	5
Hand or arm	1
**Rabies signs and symptoms^†^**	
Aerophobia (sensitivity to movement of air)	6
Anorexia	3
Anxiety	2
Fever	4
Headache	5
Hydrophobia	4
Insomnia	3
Malaise or fatigue	5
Muscle pain or spams	3
Paresthesia or localized pain	2
**Wound treatment**	
None	2
At home	2
Medical center	1
Traditional healer	1
**Received any postexposure prophylaxis**	0

* Family reported that the dog appeared normal at the time of exposure. No information was available regarding the status of the dog after 10 days.

^†^ Patients could have multiple symptoms.

The six patients resided in four of Vietnam’s 63 provinces and major cities. The patients exhibited classic signs and symptoms of rabies, including aerophobia (sensitivity to air movement) (six patients), fever (four), and hydrophobia (four). Because of cultural practices, laboratory confirmation was not available for any of the patients.

Four patients were pregnant at the time of rabies exposure, including three whose pregnancies were at 32–37 weeks’ gestation at the time of symptom onset, prompting emergency cesarean delivery. Two neonates survived, and the third died shortly after delivery from complications believed to be unrelated to rabies. The fourth pregnant patient developed rabies at approximately 18 weeks’ gestation; therefore, her fetus could not be saved.

Two other patients were breastfeeding children aged <1 year at the time of rabies exposure. Family members reported that mothers expressed concern about the possibility of PEP being transferred to their children through breast milk, fearing that the antibodies could harm the children because of their young age. Ten household contacts, including husbands of the six patients, the two children of the breastfeeding mothers, and two surviving neonates received PEP. All patients were reported to have been aware of both rabies and PEP, but none sought PEP.

Family members reported that the pregnant women were concerned about potential risks to their fetus. Only one patient, a breastfeeding mother, sought medical care after an exposure (Table), but she declined PEP after receiving wound treatment for multiple severe bites to her hand and arm by a stray dog. One of the pregnant patients was reported to have sought care from a traditional healer after being bitten twice on the leg by a stray dog. Family members reported that she had believed that treatment from a traditional healer was safer than PEP. Among the four other bite victims, one treated her wound at home with water, one with water and soap, and two did not treat their bite wound.

None of the family members reported cost or transportation as a specific deterrent to receiving PEP. The average distance from the patients’ homes to the provincial medical center where PEP was available was 32.6 miles (range = 9.7–76.4 miles) (52.4 km [range = 15.6–123.0 km]) and time to travel was 78.5 minutes (range = 23–173 minutes). The average distance to the district medical center where rabies vaccine (but not rabies immune globulin) was available was 9.8 miles (range = 3.2–18.7 miles) (15.8 km [range = 5.1–30.1 km]), with a travel time of 30.7 minutes (range = 11–65 minutes).

## Discussion

Most of the world’s estimated 60,000 annual rabies deaths occur in countries where canine rabies is endemic and where PEP is often inaccessible to bite victims (*4*). When PEP is available, documentation of vaccination hesitancy for prevention of rabies is rare. This investigation identified six rabies deaths among breastfeeding or pregnant women. Based on information provided by family members, these deaths might have been associated with unfounded concerns about vaccine-associated risks to the fetus or breastfed child. A previous U.S. report also documented refusal to receive PEP by a pregnant woman with a potential rabies exposure because of concerns about the effect of PEP in the fetus; that patient did not develop rabies (*5*). Studies have found no increased risk for spontaneous abortions, premature births, or fetal abnormalities among pregnant women after receiving PEP (*2*,*6*,*7*).

A growing body of literature documents peripartum rabies cases. Including the six cases reported here, case reports and a literature review found 20 documented probable or suspected peripartum rabies cases reported during the 114-year period, 1902–2016 (*8*,*9*). A total of 17 neonates survived, and were reported to be healthy, including eight who did not receive vaccine or immunoglobulin after caesarean or vaginal delivery (*8*). Among the three neonates who did not survive, one acquired rabies, and the other two died from complications unrelated to rabies (*8*,*10*).

Vietnam has made progress in reducing human rabies deaths. The number of human rabies cases declined 82%, from 505 cases in 1994 to 91 in 2016 (*3*). The expansion of PEP centers in the country has played a critical role in this reduction by increasing access to PEP. National PEP surveillance data indicate that an average of 400,000 vaccine doses are administered and 32,000 persons receive rabies immune globulin each year (*3*). However, work is needed to understand and address the barriers to rabies treatment among pregnant and breastfeeding women. In this investigation, only one of the six pregnant or breastfeeding bite victims sought medical care after an exposure. 

One strategy aimed at improving health care–seeking behavior and PEP acceptance is training village health workers to educate community members, including pregnant and breastfeeding women, regarding treatment options for rabies and the safety of PEP. Village health workers are respected in the community and are often sought for health advice on matters including prenatal nutrition and childhood illness. These health workers could also alert health authorities about animal exposures, prompting a community investigation to identify additional exposures. Educating traditional healers about rabies, the importance of PEP, and legal ramifications of rabies treatment by traditional healers might prevent additional cases through proper medical treatment. All six dogs associated with these cases were unvaccinated or had an unknown vaccination history, suggesting a need for improvement in the canine rabies vaccination programs in Vietnam.

Although PEP is viewed favorably by the general population in Vietnam, these findings suggest that some community members lack knowledge about its safety and appropriateness for pregnant and breastfeeding women. Given that rabies is almost always fatal in unvaccinated persons, there is an urgent need for public health messaging about the safety and effectiveness of PEP, including for pregnant and breastfeeding women.

**Summary**

**What is already known about this topic?**

Human rabies deaths are preventable through prompt administration of postexposure prophylaxis (PEP) with rabies immune globulin and rabies vaccine after exposure to rabid animals. Rabies PEP is safe for use among pregnant and breastfeeding women; studies have found no increased risk for spontaneous abortions, premature births, or fetal abnormalities among pregnant women after receiving PEP.

**What is added by this report?**

During 2015–2016, six probable cases of rabies were reported among pregnant and breastfeeding women in Vietnam. None of the six patients sought PEP after exposure. In each case, families reported the patients’ fear of risk to the fetus or breastfed child as the primary barrier to the women receiving PEP.

**What are the implications for public health practice?**

One strategy aimed at improving health care–seeking behavior and PEP acceptance is training village health workers to educate community members, including pregnant and breastfeeding women, regarding treatment options for rabies and the safety of PEP. As more countries expand access to PEP, special attention should focus on addressing vaccine hesitancy, particularly among pregnant and breastfeeding women, and improving education of community health workers.

## Footnotes

* A probable case of rabies is defined as the occurrence of an acute neurological syndrome dominated by forms of hyperactivity or paralytic syndromes progressing toward coma and death, usually by cardiac or respiratory failure, typically within 7–10 days after the first sign, in a patient with a history of animal contact.
